# Novel insights into the role of ferroptosis in temporomandibular joint osteoarthritis and knee osteoarthritis

**DOI:** 10.7150/ijms.107057

**Published:** 2025-04-09

**Authors:** Yuxin Zhang, Dahe Zhang, Xiaoyu Liao, Qingyu Xu, Lingtong Bu, Jisi Zheng, Pei Shen, Chi Yang

**Affiliations:** 1 Department of Oral Surgery, Shanghai Ninth People's Hospital, Shanghai Jiao Tong University School of Medicine; College of Stomatology, Shanghai Jiao Tong University; National Center for Stomatology; National Clinical Research Center for Oral Diseases; Shanghai Key Laboratory of Stomatology; Shanghai Research Institute of Stomatology; Research Unit of Oral and Maxillofacial Regenerative Medicine, Chinese Academy of Medical Sciences, Shanghai 200011, China.; 2 Shanghai Key Laboratory of Orthopedic Implants, Shanghai Ninth People's Hospital, Shanghai Jiao Tong University School of Medicine, Shanghai 200011, China.; 3 Department of Rehabilitation Medicine, the Affiliated Suzhou Hospital of Nanjing Medical University, Suzhou Municipal Hospital, Suzhou 215008, China.

**Keywords:** ferroptosis, temporomandibular joint osteoarthritis, knee osteoarthritis, mechanism, iron

## Abstract

Osteoarthritis (OA) is a prevalent degenerative joint disease characterized by pain, limited movement, and joint stiffness, significantly impacting the quality of life and imposing substantial economic burdens. This review paper delves into the novel insights of ferroptosis, an iron-dependent form of cell death associated with lipid peroxidation, in the context of temporomandibular joint osteoarthritis (TMJ OA) and knee osteoarthritis (KOA). We explore the pathogenic characteristics of OA, including synovitis, chondrocyte death, and extracellular matrix (ECM) degradation, and discuss the limitations of current therapeutic interventions. Emerging evidence suggests a significant relationship between ferroptosis and OA, with iron accumulation and lipid peroxidation observed in osteoarthritic cartilage. This review highlights the role of ferroptosis in chondrocyte malfunction and apoptosis, inflammation, and extracellular matrix breakdown, which are central to OA pathogenesis. We also discuss potential therapeutic targets, such as Transient Receptor Potential Vanilloid 1 (TRPV1), Glutathione Peroxidase 4 (GPX4), and Nuclear Factor Erythroid 2-Related Factor 2 (NRF2), which modulate ferroptosis and OA progression. The paper consolidates studies on ferroptosis in OA, offering a comprehensive understanding of its role and the development of innovative therapies targeting this cell death mechanism to improve treatment outcomes for OA patients.

## Introduction

More than 22 percent of adults over the age of 40 suffer from osteoarthritis (OA), a degenerative joint disease which causes pain, limited movement, as well as stiffness in the affected joints 1-3. Aside from negatively impacting people's quality of life, this ailment also places a heavy financial strain on families and society as a whole 1-3. The pathogenic characteristics of OA include synovitis, chondrocyte death, deterioration of the extracellular matrix (ECM), subchondral osteosclerosis, and osteophyte formation 4, resulting in elevated incidence and disability rates 5. Approximately 240 million persons globally are afflicted with symptomatic OA, with a prevalence incidence of 70% to 90% among those over 75 years of age 6,7. It is estimated that global case numbers for each site of OA will increase by 48.6% to 95.1% between 2020 and 2050 8, underscoring the pressing need for better therapies 9,10. Current conservative interventions for OA, such as physical workouts and oral nonsteroidal anti-inflammatory drugs (NSAIDs), seek to mitigate the disease's development during its early to mid-stages 11. Nonetheless, these therapies possess drawbacks, notably the considerable toxicity linked to NSAIDs, which may result in gastrointestinal complications and reduced renal blood flow 12,13. In cases when patients are resistant to NSAIDs, intra-articular corticosteroid injections are contemplated; nevertheless, their use is contentious owing to the possibility of hastening cartilage degeneration 14.

In this context, there is increasing interest in elucidating the significance of ferroptosis, a newly founded mechanism of cell death, exhibiting morphological characteristics such as mitochondrial shrinkage and abnormalities in cristae 15. Ferroptosis has been associated with multiple disciplines, including cardiology 16, hepatology 17, nephrology 18,19, oncology 20, and musculoskeletal research 21. Recent studies have underscored its significant relationship with OA, demonstrating iron accumulation and lipid peroxidation in osteoarthritic cartilage 22,23. The possible involvement of ferroptosis in the pathogenesis of OA is complex. Lipid peroxidation mediated by ferroptosis may result in chondrocyte malfunction and apoptosis, hence hastening cartilage deterioration 19. Elevated inflammatory markers in OA may trigger ferroptosis 24, hence perpetuating a detrimental cycle that intensifies joint inflammation and cartilage degradation 25. Ferroptosis may further affect the production of inflammatory factors in synovial fibroblasts, hence hastening the breakdown of the ECM of chondrocytes 26. Considering the pivotal function of ferroptosis in OA, targets such as transient receptor potential vanilloid 1 (TRPV1) 27,28, glutathione peroxidase 4 (GPX4) 29, nuclear factor erythroid 2-related factor 2 (NRF2) 30, acyl-CoA synthetase long-chain family member 4 (ACSL4) 31, and nuclear coactivator adaptor 4 (NCOA4) 32 have surfaced as prospective regulators of ferroptosis and modulators of OA advancement. Ferroptosis is an iron-dependent mechanism of controlled cell death marked by the formation of lipid hydroperoxides 33,34. The significance of iron buildup in inflamed tissue, resulting in cellular toxicity, in autoimmune and autoinflammatory illnesses associated with arthritis, such as OA, remains ambiguous 35. The hypoxic environment and the promotion of angiogenesis by hypoxia-inducible factors indicate that oxidative stress may significantly influence synovial tissue 36. The correlation between inflammation and ferroptosis has been investigated, since ferroptosis is associated with the production of pro-inflammatory chemicals, including IL-1β and IL-18 37, positioning it as a possible therapeutic target for arthritis-related disorders.

Articular cartilage, mostly consisting of chondrocytes and ECM, is the primary locus of basic aetiology of OA 38. Pro-inflammatory cytokines activate chondrocytes to release catabolic factors, which impede the production of COL2, proteoglycans, and other synthetic metabolic components, hence facilitating chondrocyte catabolic processes and the degradation of articular cartilage 39,40. Chronic inflammation, oxidative stress, and ferroptosis are thought to induce chondrocytes to excessively generate catabolic factors, resulting in the degradation of chondrocytes and ECM, hence accelerating the degeneration of articular cartilage in OA 41-46. Ferroptosis is characterised by increased reactive oxygen species (ROS), lipid peroxidation, iron accumulation, as well as depletion of glutathione (GSH) 47, and it has been seen in chronic bone disorders, such as OA 48-50. The buildup of ROS resulting in lipid peroxidation and initiation of ferroptosis in chondrocytes aggravates OA. Augmenting the body's antioxidant capacity and removing surplus ROS may constitute an effective treatment strategy for addressing OA. This review seeks to consolidate ferroptosis studies in OA, providing insights into the targeting of its critical elements for innovative OA therapies.

## Ferroptosis

### Definition and characteristics of ferroptosis

Ferroptosis, introduced by Dixon in 2012, is an iron-dependent form of programmed cell death (PCD) that primarily impacts cellular organelle structures, particularly mitochondrial architecture, and is marked by the buildup of lipid peroxides 51,52. The morphological characteristics include rupture of mitochondrial cristae, decrease or absence of mitochondria, degradation of the mitochondrial outer membrane, and mitochondrial shrinkage 53,54. The metabolic features include iron overload, ROS buildup, and the inactivation of the antioxidant enzyme GPX4 51,55.

### Molecular mechanisms of ferroptosis

#### The role of GPX4 and system Xc-/GSH/GPX4 regulatory axis in ferroptosis

**Figure 1:**
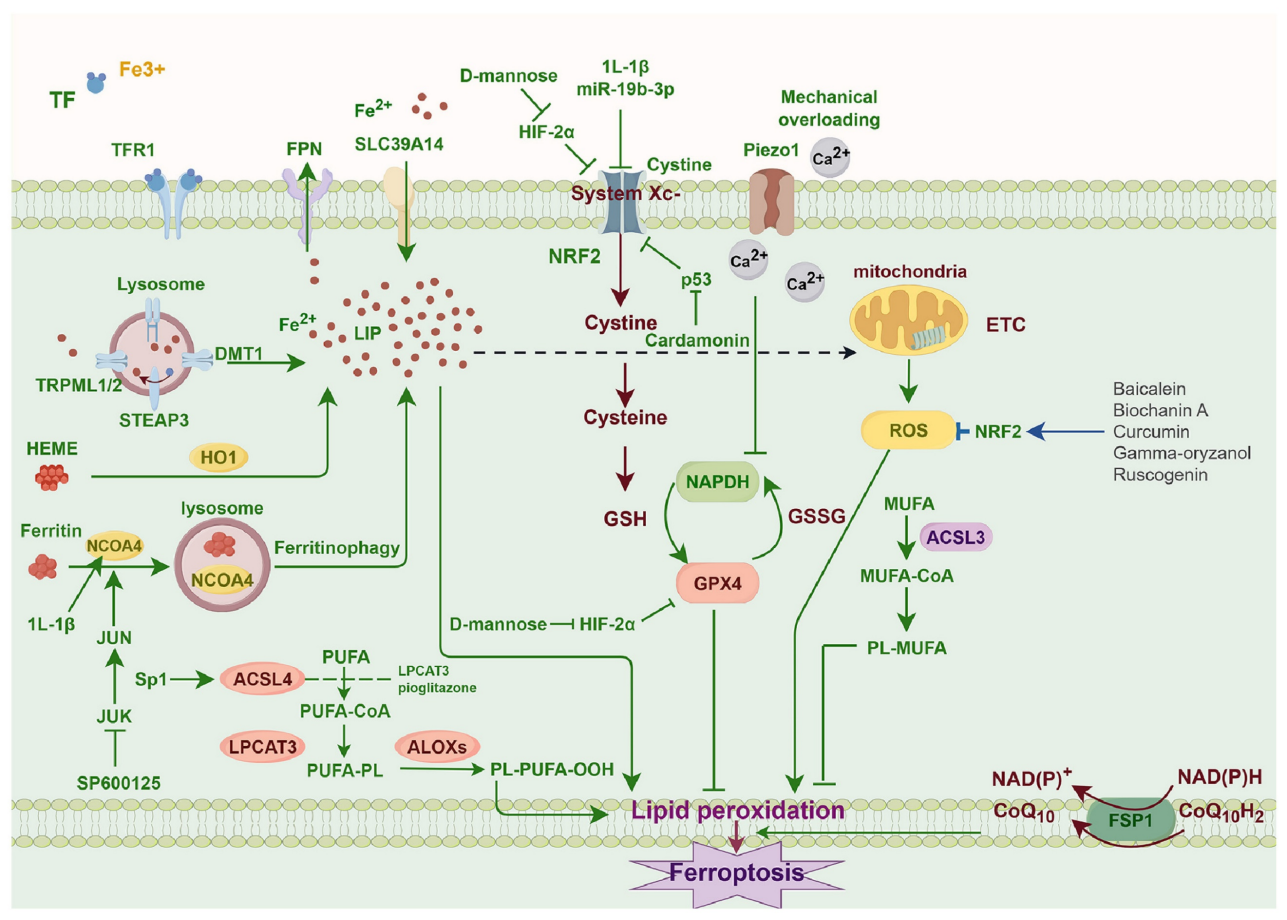
Molecular mechanisms of ferroptosis in chondrocytes.

The inactivity of the lipid repair enzyme GPX4 may result in elevated levels of lipid-based ROS, hence triggering ferroptosis 56. The system Xc-/GSH/GPX4 regulatory axis is regarded as the classical antioxidant regulatory axis of ferroptosis. The system Xc- is an upstream molecule in the axis, functioning as a sodium-independent reverse transporter. The primary function of it is to transport intracellular glutamate and extracellular cystine in a 1:1 ratio 57. Cystine is transformed into cysteine (Cys) by a redox reaction upon entering the cell, then condensing with glutamic acid (Glu) and glycine (Gly) to produce GSH 58. The body facilitates the conversion of oxidised glutathione (GSSG) to reduced GSH via GPX4, therefore safeguarding cells against peroxidative damage 59. Although the GPX4 system is the primary route, it is not the exclusive mechanism; GPX4-independent pathways, including FSP1/CoQ10, DHODH, and GCH1/BH4, all contribute significantly 56,60-62** (Figure 1)**.

#### Lipid peroxidation in ferroptosis

Lipid peroxidation principally involving the oxidative degradation of polyunsaturated fatty acids (PUFAs) in membrane phospholipids, in conjunction with reactive oxygen species. It may occur by enzymatic mechanisms involving lipoxygenases (LOXs), cyclooxygenases (COXs), and ACSL4, or through nonenzymatic processes driven by free radicals 63. One of them, ACSL4, is involved in lipid metabolism and may catalyse the conversion of PUFAs; this process can cause ferroptosis and an overabundance of lipid ROS 64. Lipid radicals react with oxygen molecules to shape lipid peroxide radicals (LOO•) and lipid hydroperoxides (LOOH), which in turn obtain hydrogen atoms from other PUFAs. This chain reaction continues until oxidative products like hydroxyl radicals (OH•) and hydrogen peroxide (H_2_O_2_) are formed. Lipid peroxidation of PUFAs is characterised by a cascade reaction because LOO• may react with PUFAs continually. Peroxidation compounds including lipid peroxide (LPO) and malondialdehyde (MDA) may be produced when PUFAs degrade 65. In addition to destroying the cell membrane's fluidity and stability, LOOH may enhance the membrane's permeability, which in turn causes lipid membrane rupture and, eventually, ferroptosis 63,66,67.

#### Iron overload in ferroptosis

A further hallmark of ferroptosis is the accumulation of iron. In physiological settings, iron primarily engages in biological activities like oxygen transport, adenosine triphosphate (ATP) production, and immunological modulation 68,69. The regulation of iron balance is mostly achieved by the synthesis and release of iron homeostasis regulators by hepatocytes, such as transferrin receptor (TFR), ferritin, and ferroportin (FPN) 68-70. In pathological settings, iron excess elevates the generation of oxygen free radicals, which subsequently exacerbates the oxidation and destruction of many cellular components 71.

#### Role of mitochondria in ferroptosis

The current dominant opinion maintains that mitochondria are involved in ferroptosis and create ROS, since mitoROS is primarily produced by oxidative phosphorylation complexes 72. MitoROS have the potential in causing mitochondrial membrane lipid peroxidation 73. This is corroborated by the fact that lipid-ROS first localised to the mitochondrial distribution in ferroptosis triggered by cystine deprivation and erastin 70. Mitochondria not only promote ferroptosis via generation of ROS but also appear to inhibit it through consuming glutamate, counteracting the suppressive influence from elevated extracellular glutamate upon the Xc system 74. The causal link between mitochondrial morphological alterations and ferroptosis remains ambiguous, despite the tight association of mitochondria with ROS generation and iron metabolism 59,70,75.

## The role of ferroptosis in temporomandibular joint osteoarthritis (TMJ OA) and knee osteoarthritis (KOA)

### The pathogenic role of ferroptosis in TMJ OA and KOA

OA is a chronic degenerative condition increasingly acknowledged for its intricate pathophysiology, which encompasses genetic, biomechanical, and metabolic components. A notable aspect that has lately garnered attention is ferroptosis, an iron-dependent variant of PCD that differs from apoptosis and other cell death modalities 76. This review seeks to encapsulate the contemporary comprehension of ferroptosis's function in the aetiology of OA, emphasising its metabolic control and the ramifications for treatment strategies.

Ferroptosis is defined by the buildup of lipid peroxides, causing the breakdown of plasma and organelle membranes, finally lead to cellular death 77. Redox-active iron significantly accelerates lipid peroxidation events, whereas the glutathione-dependent antioxidant defence system, especially GPX4, is essential in inhibiting lipid peroxidation 78. The cystine/GSH/GPX4 system is a conventional method for inhibiting iron removal, with system Xc-, being vital for the production of GSH, a key antioxidant defence route 78. The beginning and advancement of ferroptosis are primarily governed by lipid ROS, ferrous iron, and GPX4 activity, where increased ferrous iron levels and reduced GPX4 activity signify the commencement of ferroptosis 79.

**Figure 2:**
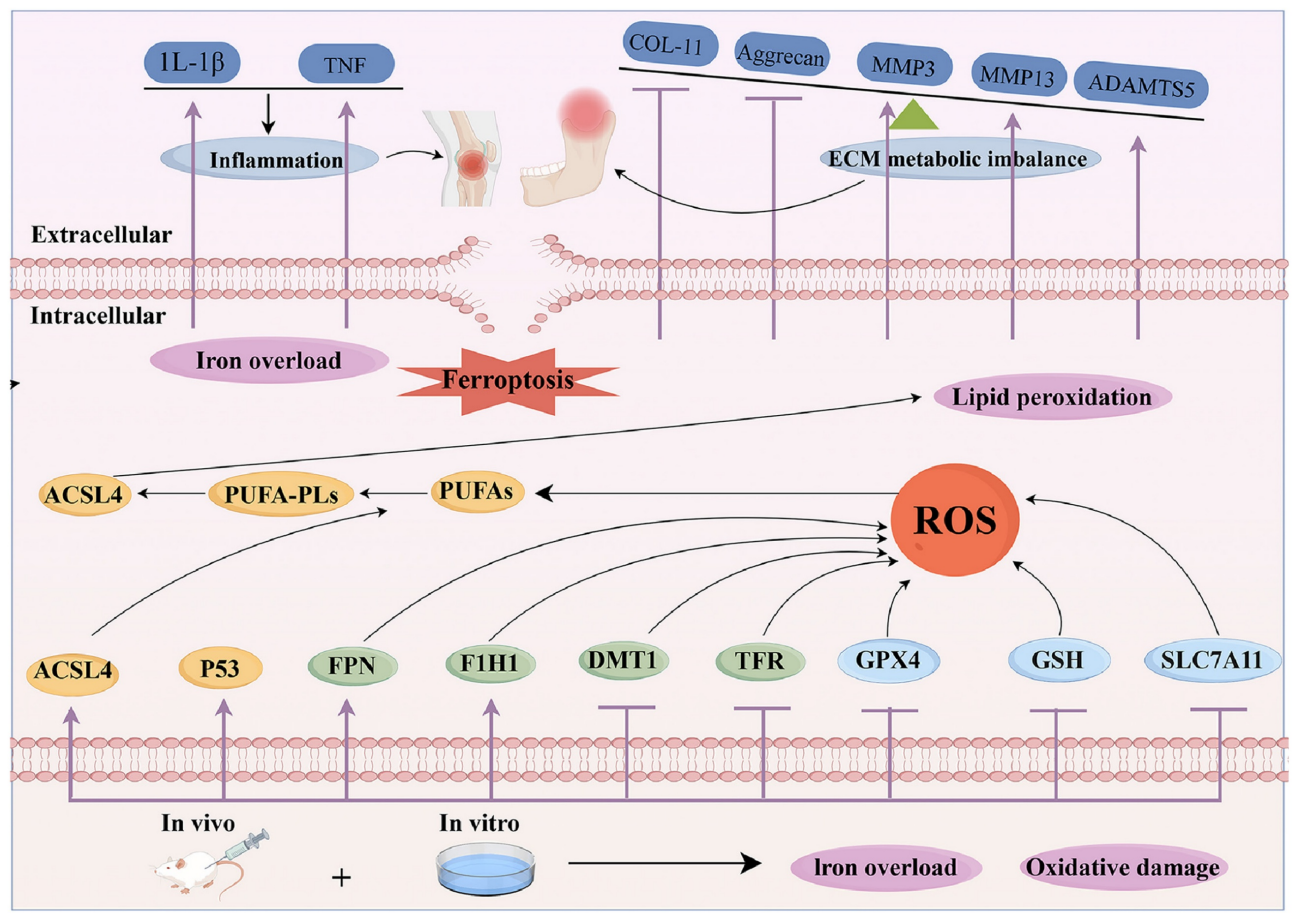
The mechanism for ferroptosis in OA.

Ferroptosis has been associated with reduced chondrocyte activity in OA, with new studies investigating the pathogenic pathways leading to chondrocyte mortality in this situation 76. The tumour suppressor gene p53 inhibits the expression of SLC7A11, leading to reduced GSH production and the suppression of GPX4. It eventually induces ferroptosis in many cell lines and maybe in chondrocytes as well 80. Inflammatory cytokines, including IL-1β/TNF-α, stimulate the expression of transferrin receptors, enhancing iron absorption and buildup, hence facilitating ferroptosis in inflammatory contexts 81. Disruption of mitochondrial membranes elevates cytoplasmic iron concentrations and impairs oxidative phosphorylation, leading to the production of ROS and exacerbating iron-induced cell death 81. Research indicates that therapies using the iron chelator deferoxamine (DFX) may alleviate the advancement of OA, underscoring the significance of iron in the condition 82. Hypoxia-inducible factor 2α (HIF-2α) was shown to significantly influence cartilage formation, the development of OA, and cellular resistance to ferritin 83. Investigations applying IL-1β to stimulate chondrocytes within the OA environment have shown an up-regulation of HIF-1α and LPO levels, with a down-regulation of GSH levels in chondrocytes subjected to IL-1β stimulation 84. Patients with OA exhibit elevated iron levels in synovial tissue, and the impairment of iron homeostasis-regulating genes causes cellular iron accumulation, resulting in heightened production of ROS as well as the increased level of collagen II and MMP13, which are established hallmarks of OA 85 **(Figure 2)**.

**Figure 3:**
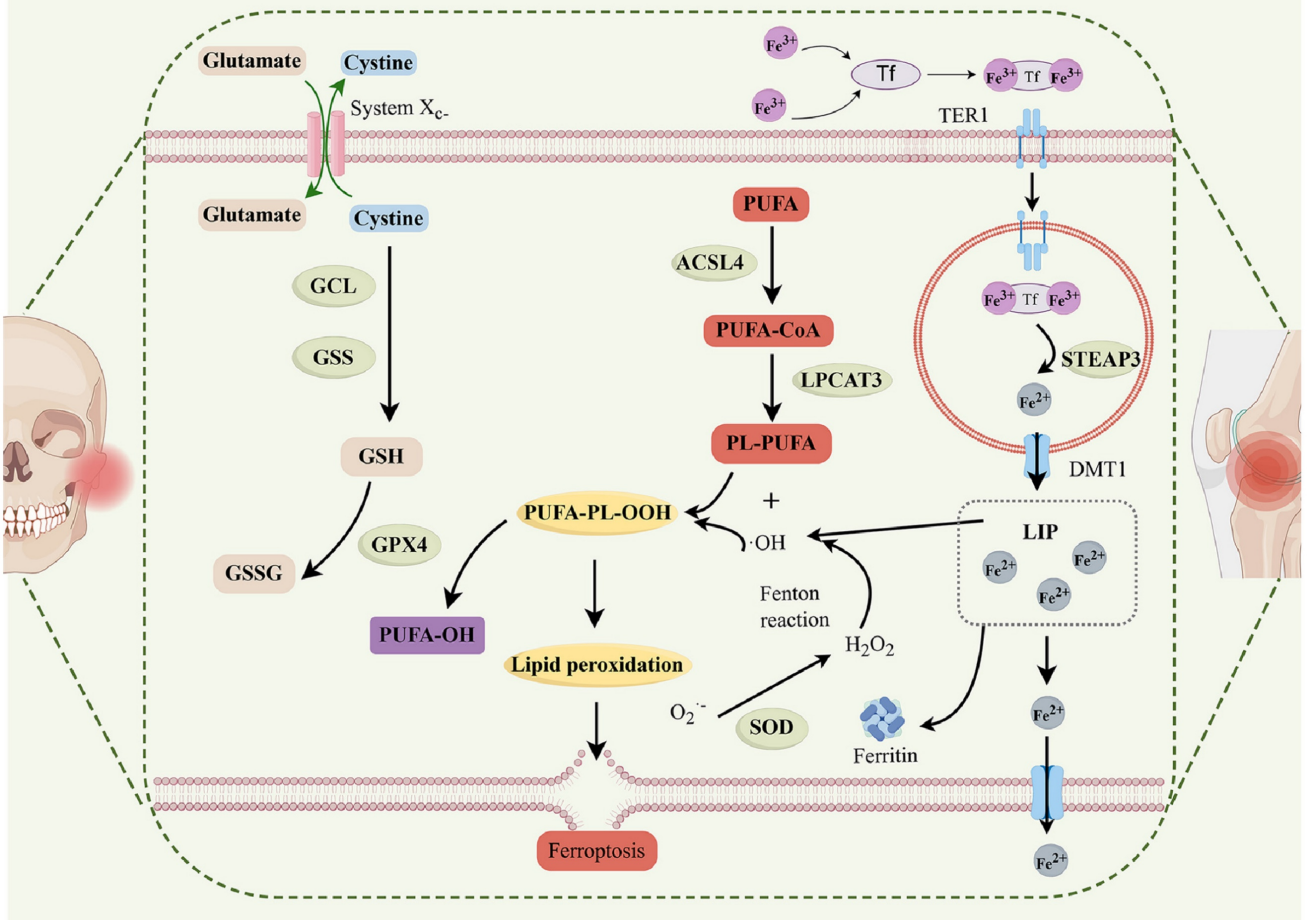
Ferroptosis in TMJ OA and KOA. ACSL4, acylCoA synthetase long-chain family member 4; DMT1, ferrous ion membrane transport protein DMT1; LIP, intracellular labile iron pool; FPN, ferroportin; LPCAT3, lysophosphatidylcholine acyltransferase 3; GCL, glutamate cysteine ligase; GPX4, glutathione peroxidase 4; GSH, glutathione; GSS, glutathione synthetase; GSSG, oxidized glutathione; PUFA, polyunsaturated fatty acid; PL, phospholipid; STEAP3, metalloreductase STEAP3; SOD, superoxide dismutase; Tf, transferrin; TFR1, transferrin receptor protein 1.

The pathogenic mechanism of OA include damage to periarticular cartilage and subchondral bone, synovitis, and the critical involvement of chondrocytes in this process. In individuals with OA, the iron content in synovial fluid is elevated, and greater serum ferritin levels correlate with increased radiographic severity of the joints compared to healthy controls 86,87. Chondrocyte ferroptosis facilitates the advancement of OA, as shown by both *in vivo* and *in vitro* studies 88. Crucial enzymes, including ACSL4 and LPCAT3, are essential to the biosynthesis of PUFA-Pls, which serve as substrates for LPO 89,90. The cumulative consequences of these processes induce lipid peroxidation, eventually causing ferroptosis of chondrocytes in OA **(Figure 3)**.

Using high-resolution mass spectrometry, Zou *et al.* completed a thorough analysis of the protein expression patterns in synovial fluid of patients with temporomandibular joint (TMJ) anterior disc displacement (ADD) 91. The research revealed notable elevations in proteins including FGL2, THBS4, and TNC in ADD without OA, signifying active ECM and collagen metabolism, whereas FGFR1, FBLN2, and GRB2 were heightened in ADD with OA, implying a role in ferroptosis. Moreover, proteins such as P4HB and CBLN4 exhibited persistent overexpression throughout the development of the illness. These results elucidate the molecular processes of TMJ OA and provide possible treatment targets 91. Ferroptosis is essential in chondrocytes and is more susceptible to cellular damage due to heightened buildup. This vulnerability is linked to inflammatory mediators like IL-1β and IL-6 that were shown to trigger ferroptosis in OA. The exact mechanism of ferroptosis in OA, coupled with discovery of possible targets in the ferroptosis pathway, is essential to improve therapeutic approaches for persons suffering from OA.

### The ferroptosis-based strategies in TMJ OA and KOA

**Figure 4:**
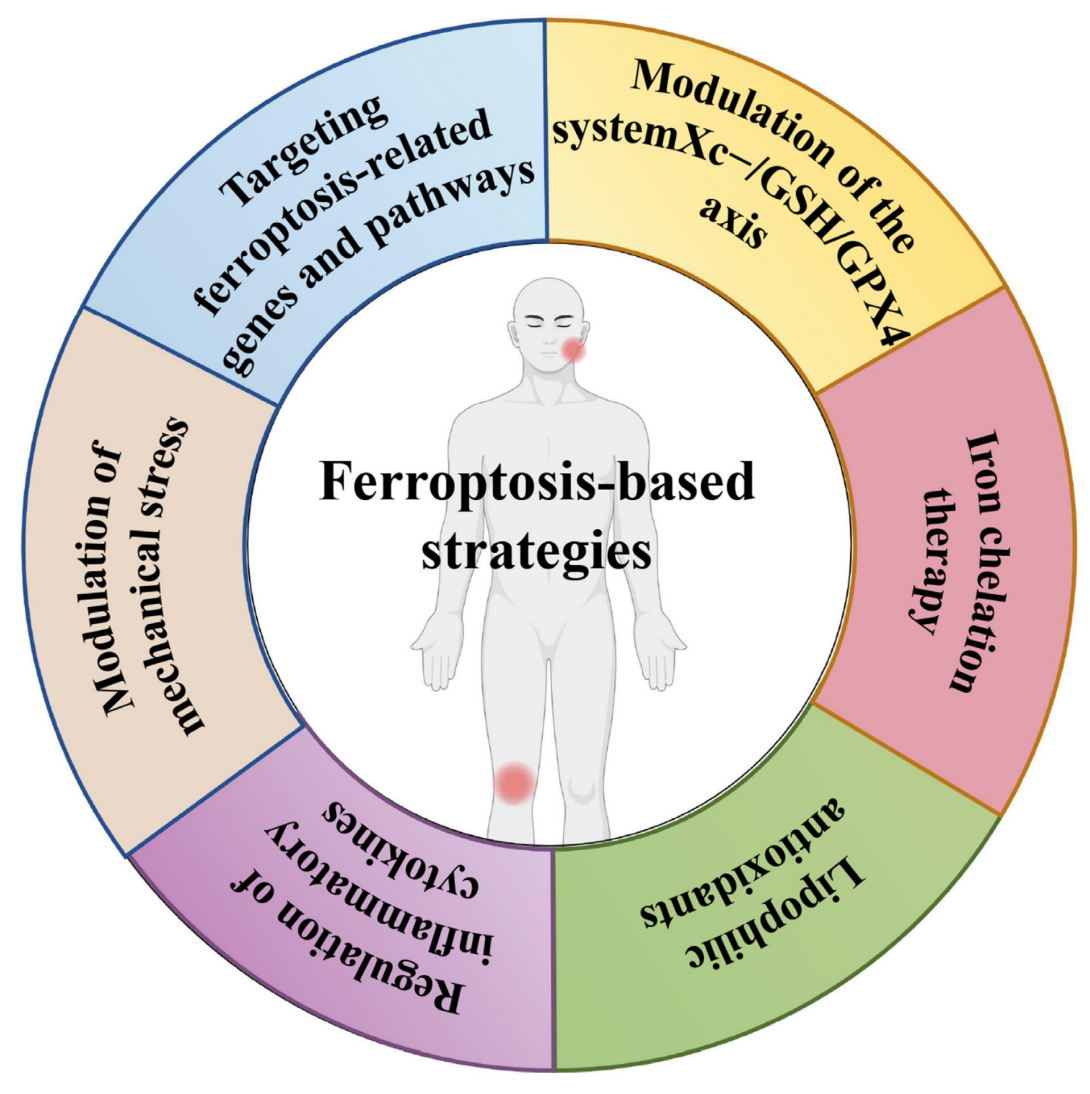
The ferroptosis-based strategies in TMJ OA and KOA.

OA is marked by cartilage deterioration, synovial inflammation, and alterations in subchondral bone. Despite comprehensive research, effective therapies for OA remain constrained, especially in preventing or reversing disease progression. Recent studies have emphasised the significance of ferroptosis, a kind of PCD characterised by iron-dependent lipid peroxidation, in the aetiology of OA. This review encapsulates the current comprehension of ferroptosis in OA and examines prospective treatment approaches aimed at this pathway **(Figure 4)**.

#### Ferroptosis and chondrocyte death

Chondrocytes, the intrinsic cells of cartilage, are essential for preserving cartilage homeostasis. In OA, chondrocytes experience substantial alterations, including senescence, apoptosis, and necrosis. Ferroptosis has become a notable factor in chondrocyte apoptosis in OA. Recent studies indicate that ferroptosis indicators, including GPX4 and SLC7A11, are reduced in osteoarthritic chondrocytes, although lipid peroxidation and iron buildup are elevated 92,93. These alterations indicate that ferroptosis plays a significant role in chondrocyte apoptosis and cartilage deterioration in OA.

#### Ferroptosis-related genes and pathways

Ferroptosis regulation in osteoarthritic chondrocytes has been linked to various genes and mechanisms. The system Xc-/GSH/GPX4 regulation axis, crucial for sustaining cellular redox equilibrium, is impaired in OA. The downregulation of SLC7A11, a component of system Xc-, results in diminished GSH production and GPX4 activity, hence increasing chondrocyte vulnerability to ferroptosis 92,93. Moreover, miRNAs like miR-19b-3p have shown the ability to induce ferroptosis in osteoarthritic chondrocytes by targeting SLC7A11 92. Pang *et al.* examine the function of GPX4 and ferroptosis in the advancement of TMJ OA 94. The authors show, via the examination of patient samples and animal models, that ferroptosis is triggered in TMJ OA tissues and synovial fluid, as indicated by elevated levels of ferroptosis markers and diminished antioxidant system function. GPX4 expression is significantly reduced in TMJ OA, facilitating the induction of ferroptosis 94. The research indicates that the inhibition of ferroptosis, either by GPX4 overexpression or the use of ferroptosis inhibitors, markedly improves the pathological alterations in TMJ OA in rat models. Overexpression of GPX4 enhances cell viability by suppressing ferroptosis and increasing the activity of the antioxidant system. The results suggest that ferroptosis and GPX4 targeting might be an innovative treatment approach for the therapy of TMJ OA 94. Additionally, inflammatory cytokines, including IL-1β, have been shown to induce ferroptosis in chondrocytes. Treatment with IL-1β enhances iron absorption, ROS generation, and lipid peroxidation, concurrently reducing GPX4 expression 95. These alterations result in chondrocyte apoptosis and cartilage deterioration. The regulation of ferroptosis is influenced by several variables, including EAAT1, a membrane protein, and HSPA5, a member of the heat shock protein A family. Inhibiting IL-1β-induced chondrocyte ferroptosis, overexpressing HSPA5 activates the glutathione system, and upregulating EAAT1 suppresses ferroptosis and slows the course of OA, according to previous research 96,97.

The effects of ferroptosis in the fibrocartilage of the TMJ and the hyaline cartilage of the knee exhibit both similarities and potential differences, which can be attributed to the distinct tissue characteristics and microenvironments of these two joint types. Firstly, ferroptosis, an iron-dependent form of PCD characterized by the accumulation of lipid peroxides, plays a pathogenic role in both TMJ OA and KOA. In both conditions, ferroptosis contributes to chondrocyte death and cartilage degeneration. This is evidenced by reduced expression of ferroptosis-inhibiting genes such as GPX4 and SLC7A11, along with increased lipid peroxidation and iron accumulation in osteoarthritic chondrocytes from both the TMJ and the knee. However, there are some potential differences in the effects of ferroptosis between these two types of cartilage. Fibrocartilage, which is the primary tissue type in the TMJ, has a unique composition and structure compared to hyaline cartilage found in the knee. Fibrocartilage contains a higher proportion of type I collagen and is more fibrous, which may influence its susceptibility to ferroptosis and the subsequent disease progression. Additionally, the mechanical stress experienced by the TMJ and the knee differs, with the TMJ being subjected to complex movements and loads during jaw function, while the knee experiences primarily compressive and shear forces. These differences in mechanical stress may modulate the ferroptosis pathway in distinct ways, affecting chondrocyte metabolism and survival. Furthermore, the inflammatory milieu in TMJ OA and KOA may also contribute to differential effects of ferroptosis. Inflammatory cytokines, such as IL-1β, have been shown to induce ferroptosis in chondrocytes. However, the specific inflammatory profiles and cytokine levels may vary between the TMJ and the knee, leading to distinct outcomes in terms of ferroptosis-mediated chondrocyte death and cartilage damage. Collectively, while ferroptosis plays a significant role in the pathogenesis of both TMJ OA and KOA, the specific effects may differ due to the distinct tissue characteristics, mechanical stress, and inflammatory environments of TMJ and knee. Further research is needed to elucidate these differences and to develop targeted therapeutic strategies that address the unique aspects of ferroptosis in each joint type.

#### Therapeutic strategies targeting ferroptosis in OA

##### Modulation of the system Xc-/GSH/GPX4 axis

Numerous treatment approaches have focused on modifying the system Xc-/GSH/GPX4 axis because of its significance in controlling ferroptosis. By stimulating the system Xc-/GSH/GPX4 axis, for instance, it has been shown that traditional Chinese medicines (TCMs) prevent ferroptosis in OA chondrocytes. A substance called icariside II, which comes from epimedium, has been shown to repress ferroptosis-related gene expression in chondrocytes, activate the GSH/GPX4 axis, and reduce the production of iron transporter proteins 98. Similarly, by upregulating GPX4 and SLC7A11 expression in chondrocytes, baicalein, an extract from scutellaria baicalensis, has been shown to prevent ferroptosis and postpone the development of OA 99. A further research examines the contribution of chondrocyte ferroptosis to the development of TMJ OA and the possibility of liproxstatin-1 (Lip-1) as a treatment to reduce cartilage deterioration 100. Utilizing a variety of animal models of TMJ OA caused by unilateral anterior crossbite (UAC), occlusion disorder (OD), monosodium iodoacetate (MIA), and IL-1β, the study shows that ferroptosis, which is typified by elevated p53 and ACSL4 and decreased GPX4, is a major contributor to TMJ OA.

A potential treatment strategy for TMJ OA could be the injection of the ferroptosis inhibitor Lip-1, which successfully reduced chondrocyte ferroptosis, restored mitochondrial function, decreased ROS and lipid peroxidation, and improved the integrity of the cartilage matrix 100. In their study, Guo *et al.* detail the steps used to create and characterize PCA NPs, which are nanoparticles made from the naturally occurring phenolic acid p-coumaric acid, with the aim of treating TMJ OA 101. *In vitro* investigations showed that PCA NPs were more effective than hyaluronic acid at reducing inflammation and oxidative damage. Injecting PCA NPs into the joints of TMJ OA-affected rats slowed the disease's course over the long term by improving cell proliferation and matrix formation, decreasing inflammation and oxidative stress, and chondrocyte ferroptosis 101. This study's results demonstrate that PCA NPs have therapeutic potential by addressing several TMJ OA pathological pathways. By influencing important markers associated with ferroptosis, including GPX4, ACSL4, and SLC7A11, PCA NPs were discovered to suppress chondrocyte ferroptosis, a newly defined cell death pathway involved in the advancement of OA. This comprehensive approach positions PCA NPs as a viable choice for the formulation of novel therapeutic techniques for the treatment of TMJ OA 101. He *et al.* examine the preventive benefits of Biochanin A (BCA) against KOA produced by iron overload 102. *In vivo* and *in vitro* tests revealed that BCA markedly decreased iron accumulation in the knee joints of mice with iron overload, therefore mitigating the severity of KOA. Moreover, BCA was shown to impede chondrocyte ferroptosis via modulating iron homeostasis, diminishing ROS buildup, and activating the Nrf2/system XC-/GPX4 signaling pathway, which combined facilitated its chondroprotective effects 102. The study emphasizes the possible therapeutic benefits of BCA in treating KOA related to iron excess. BCA exhibits a unique method of action by directly reducing intracellular iron levels and alleviating oxidative stress, perhaps paving the way for new therapeutic options for this illness. The study's results elucidate the molecular pathways that underlie BCA's preventive actions against iron overload-induced KOA.

##### Iron chelation therapy

Iron accumulation is a prevalent characteristic in OA and facilitates ferroptosis-mediated chondrocyte apoptosis. Consequently, iron chelation treatment has been suggested as a viable therapeutic approach. Deferoxamine (DFO), an iron chelator, were shown the ability to suppress ferroptosis among osteoarthritic chondrocytes by diminishing iron buildup and lipid peroxidation 103. Intra-articular administration of DFO has been shown to impede the development of OA in murine models 103. These results indicate that iron chelation therapy might be a feasible treatment for OA by addressing ferroptosis.

##### Lipophilic antioxidants

Lipophilic antioxidants, including ferrostatin-1 (Fer-1), have shown the ability to impede ferroptosis by neutralizing lipid peroxides. Fer-1 has been shown to counteract ferroptosis-induced chondrocyte mortality and cartilage deterioration in OA models 104,105. Guan *et al.* examine the influence of the gut microbiota metabolite capsiate on the inhibition of ferroptosis-associated OA progression 106. Through the analysis of clinical data and the execution of *in vivo* and *in vitro* tests, the researchers discovered that capsiate levels were markedly reduced in OA patients relative to healthy controls. They found that capsiate suppressed the production of HIF-1α and activated SLC2A1, thereby diminishing ferroptosis and alleviating OA in murine models 106. The research clarifies the molecular mechanism by which capsiate has a preventive effect against OA. Utilizing bioinformatics analysis, gene knockout tests, and molecular modeling, the researchers demonstrated that SLC2A1 modulated HIF-1α expression and oxidative stress levels, which are essential for ferroptosis 106. The results indicate that capsiate, a product of gut microbiota, may serve as a possible therapeutic approach for OA by targeting the gut-joint axis. The results indicate that lipophilic antioxidants might be beneficial in the treatment of OA by safeguarding chondrocytes against ferroptosis.

##### Regulation of inflammatory cytokines

Inflammatory cytokines, including IL-1β, are pivotal in triggering ferroptosis in osteoarthritic chondrocytes. Consequently, targeting these cytokines may provide a useful treatment approach. Puerarin, a substance extracted from pueraria lobata, has shown the capacity to enhance chondrocyte viability and reduce inflammatory cytokine levels in chondrocytes exposed with IL-1β 107. Gamma-oryzanol, a molecule derived from rice bran oil, has been shown to inhibit ferroptosis and cartilage degradation by engaging with the Keap1 and Nrf2 binding sites in IL-1β-treated chondrocytes 108.

##### Modulation of mechanical stress

Mechanical stress is a significant component that induces ferroptosis in osteoarthritic chondrocytes. Moderate mechanical stress could activate Nrf2/GPX4/HO-1 axis and inhibit NF-κB pathway, therefore reducing chondrocyte ferroptosis 109. Excessive mechanical stress may trigger catabolic processe in chondrocytes to facilitate ferroptosis. Consequently, regulating mechanical stress might serve as an effective therapeutic approach for addressing OA by targeting ferroptosis. Through the modulation of ferroptosis in fibroblast-like synoviocytes (FLSs), Hu *et al.* examine the therapeutic effects of Lipoxin A4 (LXA4) on the development of KOA 110. The authors show that LXA4 activates the expression of GPX4, lysophosphatidic acid receptor-3 (LPAR3), and estrogen receptor beta (ESR2) in FLSs, which inhibits ferroptosis and reduces inflammation and chondrocyte matrix breakdown. The work shows that LXA4 acts via the ESR2/LPAR3/Nrf2 axis to provide its anti-inflammatory and anti-ferroptotic actions *in vitro* cell co-culture and *in vivo* rat models, offering a novel therapeutic target for the intervention of KOA 110. The research examines the impact of exercise on KOA therapy, demonstrating that moderate-intensity treadmill exercise enhances LXA4 synthesis, which subsequently suppresses synovial ferroptosis and mitigates KOA pathogenesis. Utilizing PHTPP, a selective ESR2 antagonist, the authors demonstrate that the therapeutic benefits of exercise are largely reliant on the ESR2-mediated signaling system, therefore providing novel insights into the processes governing exercise treatment for KOA 110.

##### Targeting ferroptosis-related genes and pathways

Moreover, focusing on ferroptosis-related genes and pathways may be beneficial in the treatment of OA. Inhibition of miR-19b-3p expression, which facilitates ferroptosis by targeting SLC7A11, has shown benefit effects on chondrocytes against ferroptosis in OA 92. Likewise, the activation of TRPV1, which suppresses the ferroptosis pathway in chondrocytes, has been shown to slow the development of OA 111,112. This research conducted by Bao *et al.* examined the influence of METTL3 on the advancement of KOA via regulating ferroptosis in chondrocytes 113. The study demonstrated that METTL3 promotes ferroptosis in chondrocytes via m6A methylation of high mobility group box 1 (HMGB1), intensifying pain and cartilage deterioration in KOA model. The suppression of METTL3 mitigated these adverse effects, suggesting that the METTL3/HMGB1 axis is integral to the pathophysiology of KOA 113. The research revealed that METTL3 depletion suppressed inflammatory responses, ECM disintegration, and ferroptosis in chondrocytes, resulting in enhanced cartilage regeneration and decreased knee discomfort in KOA mice. The results indicate that focusing on the METTL3/HMGB1 pathway might provide a new therapeutic approach for treating KOA 113. The results indicate that targeting genes and pathways associated with ferroptosis could provide new options for the treatment of OA. He *et al.* examine the therapeutic benefits and underlying mechanisms of vitamin K2 (VK2) on OA 114. The study shows that VK2 substantially mitigates OA by inhibiting chondrocyte ferroptosis and the destruction of the ECM via *in vivo* and* in vitro* trials. VK2 has protective effects via dual control of GPX4, which suppresses ferroptosis and modifies ECM breakdown through the MAPK/NF-κB axis 114. The results indicate that VK2 enhances bone mass and cartilage thickness in OA rats, alleviates pain, and lowers the OARSI score. VK2 therapy enhances GPX4 expression, resulting in reduced Fe^2+^ levels, ROS, lipid-ROS generation, and mitochondrial membrane potential, hence preventing chondrocyte ferroptosis. Moreover, VK2 suppresses the MAPK/NF-κB axis, resulting in decreased production of MMP3 and MMP13, while enhancing the synthesis of type II collagen and proteoglycans, so collectively mitigating ECM breakdown 114.

##### Challenges

Notwithstanding the encouraging therapeutic prospects of targeting ferroptosis in OA, several difficulties persist. The precise function of ferroptosis in the pathophysiology of OA is inadequately understood. Additional study is required to clarify the molecular pathways that drive ferroptosis-related chondrocyte death and cartilage deterioration. The effectiveness and safety of existing ferroptosis-targeting medicines must be confirmed via clinical studies. The diversity among OA patients may restrict the efficacy of single-target treatments. Combination medicines that target various pathways may enhance the efficacy of OA treatment. Future research must concentrate on discovering new ferroptosis-related biomarkers and therapeutic targets, creating more effective and safer ferroptosis inhibitors, and assessing the effectiveness of combination therapy in clinical trials. Furthermore, comprehending the interplay between ferroptosis and other cell death mechanisms, including apoptosis and necrosis, might provide novel insights into the etiology of OA and inform the formulation of more holistic treatment approaches. Ferroptosis has emerged as an important factor in chondrocyte death and cartilage deterioration in OA. Focusing on ferroptosis-related pathways, including the system Xc-/GSH/GPX4 axis, iron metabolism, and inflammatory cytokines, may provide novel therapeutic strategies for managing OA. Further study is needed to comprehensively demonstrate the role of ferroptosis in the etiology of OA and to devise effective and safe treatment options. Ongoing improvements in ferroptosis research are anticipated to provide novel therapeutics targeting this pathway, therefore enhancing the quality of life for people with OA.

Collectively, the role of ferroptosis in OA is multifaceted and has been extensively explored in recent studies. Ferroptosis, an iron-dependent form of cell death characterized by excessive lipid peroxidation, is distinct from apoptosis and necrosis. It plays a significant role in the pathogenesis of OA, particularly in the context of chondrocyte death and cartilage degradation. Ferroptosis contributes to OA through several mechanisms: 1) Iron overload and lipid peroxidation: elevated iron levels in OA tissues lead to increased lipid peroxidation, which damages cell membranes and induces cell death. This process is exacerbated by the downregulation of protective enzymes like GPX4 and the disruption of the system Xc-/GSH/GPX4 axis. 2) Inflammatory cytokines: inflammatory cytokines such as IL-1β and TNF-α upregulate transferrin receptors, leading to increased iron uptake and accumulation, thereby promoting ferroptosis. These cytokines also downregulate GPX4, further sensitizing chondrocytes to ferroptosis. 3) Mechanical stress: excessive mechanical stress can induce ferroptosis in chondrocytes by disrupting iron homeostasis and increasing oxidative stress. Moderate mechanical stress, however, can activate protective pathways like the Nrf2/GPX4/HO-1 axis, thereby reducing ferroptosis. 4) Genetic and epigenetic regulation: certain genes and pathways, such as miR-19b-3p and the METTL3/HMGB1 axis, have been identified as key regulators of ferroptosis in OA. For example, METTL3 promotes ferroptosis through m6A methylation of HMGB1, leading to cartilage degradation and pain in OA. Causes of ferroptosis in OA includes: 1) Iron accumulation: elevated levels of iron in synovial fluid and cartilage tissues are a hallmark of OA. This iron overload can be due to disrupted iron homeostasis, increased iron absorption via transferrin receptors, or decreased iron export via ferroportin. 2) Oxidative stress: increased production of ROS and lipid peroxides due to impaired antioxidant defenses (e.g., reduced GPX4 activity) leads to oxidative damage and cell death. 3) Inflammatory mediators: inflammatory cytokines not only induce iron accumulation but also directly activate ferroptosis pathways. For instance, IL-1β can increase iron uptake and decrease GPX4 expression, thereby promoting ferroptosis. 4) Genetic and molecular pathways: dysregulation of genes involved in iron metabolism, lipid peroxidation, and antioxidant defense (e.g., SLC7A11, GPX4, Nrf2) contributes to the susceptibility of chondrocytes to ferroptosis. Ferroptosis plays a crucial role in OA by contributing to chondrocyte death, cartilage degradation, and joint inflammation. Its initiation is driven by iron overload, oxidative stress, inflammatory cytokines, and disrupted genetic pathways. Understanding these mechanisms provides valuable insights for developing targeted therapies to mitigate OA progression.

## Conclusions and future perspectives

OA is a common degenerative joint disease marked by the gradual degradation of cartilage, synovitis, and remodeling of subchondral bone. Effective therapies for OA are limited, sometimes necessitating joint replacement in late stages. Comprehending the etiology of OA is essential for formulating more efficacious therapy approaches. Recent study has emphasized the significance of ferroptosis, an iron-dependent mode of cell death induced by lipid peroxidation, in the development of OA. Research has focused on three primary domains related to ferroptosis in OA: iron homeostasis, lipid metabolism, and the antioxidant defense mechanism. Chondrocytes in OA are less viable because disturbances in iron homeostasis, such changes in the expression of proteins involved in iron metabolism, cause an increase in iron input and a decrease in iron efflux. An important factor in ferroptosis is lipid peroxidation, especially of polyunsaturated fatty acids, in which LOX and ACSL4 play essential roles. In OA, ferroptosis may be inhibited by antioxidant systems such as the NRF2 signaling pathway and the system Xc-/GSH/GPX4 system. Also involved in OA are many other forms of PCD, including autophagy, pyroptosis, apoptosis, cuproptosis, and necroptosis. Inflammatory substances, which may be produced by these processes, might worsen the symptoms of OA. The development of OA is regulated by molecules including IER1, NF-κB, Jak, and mTOR.

Subsequent research may concentrate on the following aspects: 1) Expansion of ferroptosis research: additional research into the involvement of ferroptosis in OA is essential, especially with critical regulators such as FSP1, DHODH, and GCH1, which remain unexamined in the context of OA. Research should include other cell types implicated in OA, including osteoblasts, macrophage, and synovial cells, rather than just concentrating on chondrocytes. 2) Iron homeostasis and lipid metabolism: a comprehensive knowledge of iron homeostasis and lipid metabolism in OA is essential. Investigating how variables such as IL-1β and H_2_O_2_ influence the level of iron related proteins might provide insights into illness development. Inhibiting LOX and ACSL4 activity may effectively prevent chondrocyte ferroptosis and decelerate OA development. 3) Antioxidant systems and ROS: the function of antioxidant systems in mitigating ferroptosis in OA warrants further investigation. Pharmaceuticals that stimulate these pathways may alleviate chondrocyte ferroptosis and slow the course of OA. ROS are pivotal molecules in OA, governing not only ferroptosis but also mitophagy and many kinds of autophagy. Examining ROS interaction in OA microautophagy and its modulation of ferroptosis is a viable research avenue. 4) PCD and signaling pathways: additional investigation is required to clarify the intricate interactions among several forms of PCD in OA. Comprehending the regulatory roles of molecules like as NF-κB, Jak, and mTOR in these processes may provide novel therapeutic targets. Investigating therapies that focus on PCD and signaling pathways associated with OA progression may provide more efficacious and less invasive options compared to existing treatments. 5) Therapeutic development: compounds capable of inhibiting PCD, namely ferroptosis, warrant exploration as prospective therapeutic agents for OA. The precise network processes of crosstalk governing OA ferroptosis and other types of autophagy need comprehensive investigation to pinpoint novel treatment targets. 6) Global study collaboration: additional confirmatory studies and study from a wider array of nations are important to verify results and enhance the comprehension of ferroptosis in OA. Collaborative initiatives should be promoted to bridge research gaps and expedite the development of novel therapies for OA. Collectively, ferroptosis and other types of PCD are pivotal in the course of OA. Additional investigation into these mechanisms, their control, and prospective therapeutic targets is crucial for formulating more effective therapeutics for OA. Through comprehensive experimental and clinical research, PCD may be used as a target for the development of therapeutic medications that tackle the underlying causes of OA without adverse effects or consequences.
